# Supplementary material for: Time‑resolved multi-omic analysis of paclitaxel exposure in human iPSC‑derived sensory neurons unveils mechanisms of chemotherapy‑induced peripheral neuropathy
Source: Cell Death Dis. 2026 Feb 10;17(1):211. doi: 10.1038/s41419-026-08445-2 (PMC12921266; doi:10.1038/s41419-026-08445-2)
Supplement: Supplementary file 15 — Dataset 1. [file 41419_2026_8445_MOESM15_ESM.zip › CDD_Open_Data/04_RNA_Seq/Timeline_panel_plots_etc_2023-01-23_1609.zip!/Panel_Plots_etc/Evidence_Plots/48h/evidence_plot_standard contrasts_msigdb_reactome_48h_PTX_vs_DMSO_ID5_M16227_cholesterol.pdf]

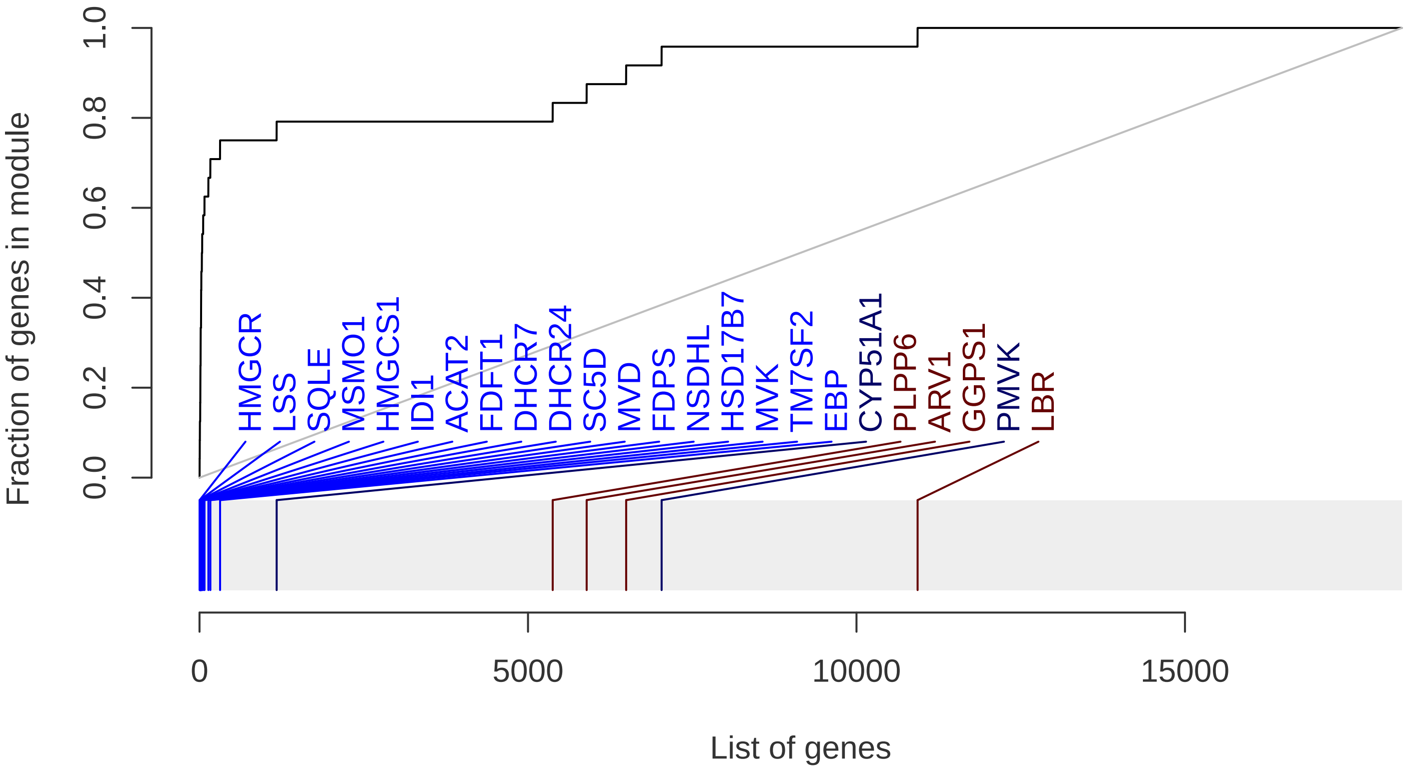

Fraction of genes in module
0.0
0.2
0.4
0.6
0.8
1.0
HMGCR
LSS
SQLE
MSMO1
HMGCS1
IDI1
ACAT2
FDFT1
DHCR7
DHCR24
SC5D
MVD
FDPS
NSDHL
HSD17B7
MVK
TM7SF2
EBP
CYP51A1
PLPP6
ARV1
GGPS1
PMVK
LBR
0
5000
10000
15000
List of genes
